# Glucose and Blood Pressure-Dependent Pathways–The Progression of Diabetic Kidney Disease

**DOI:** 10.3390/ijms21062218

**Published:** 2020-03-23

**Authors:** Devang M. Patel, Madhura Bose, Mark E. Cooper

**Affiliations:** 1 Department of Diabetes, Monash University Central, Clinical School, Melbourne, VIC 3004, Australia; madhura.bose@monash.edu; 2 Department of Endocrinology and Diabetes, The Alfred Hospital, Melbourne, VIC 3004, Australia

**Keywords:** diabetic kidney disease, diabetic nephropathy, diabetic complications, vasoactive pathways

## Abstract

The major clinical associations with the progression of diabetic kidney disease (DKD) are glycemic control and systemic hypertension. Recent studies have continued to emphasize vasoactive hormone pathways including aldosterone and endothelin which suggest a key role for vasoconstrictor pathways in promoting renal damage in diabetes. The role of glucose per se remains difficult to define in DKD but appears to involve key intermediates including reactive oxygen species (ROS) and dicarbonyls such as methylglyoxal which activate intracellular pathways to promote fibrosis and inflammation in the kidney. Recent studies have identified a novel molecular interaction between hemodynamic and metabolic pathways which could lead to new treatments for DKD. This should lead to a further improvement in the outlook of DKD building on positive results from RAAS blockade and more recently newer classes of glucose-lowering agents such as SGLT2 inhibitors and GLP1 receptor agonists.

## 1. Introduction

Diabetes mellitus, commonly referred to as diabetes, is clinically characterized by hyperglycemia due to insulin insufficiency arising from a lack of insulin production or insulin insensitivity [1]. Diabetes is associated with numerous complications. These complications are wide-ranging and are mainly caused because of chronic elevation of blood glucose levels. Elevated blood glucose causes damage to small blood vessels and arteries, known as “microvascular disease” and “macrovascular disease” respectively. Underlying vascular injury causing organ and tissue damage results in diabetic complications. These complications include neuropathy (neural damage), retinopathy (eye disease) and nephropathy (kidney disease) [2]. In this review, we will primarily focus on diabetic nephropathy. 

Diabetic nephropathy, more commonly known as diabetic kidney disease (DKD), remains a major cause of morbidity and mortality in both type 1 diabetes (T1D) and type 2 diabetes (T2D). Diabetic subjects have an elevated risk of DKD, with features of DKD developing in approximately half of all patients with T2D and one-third with type 1 diabetes. DKD is clinically defined by the presence of impaired renal function and/or elevated urinary albumin excretion and is the main cause of the end-stage renal disease (ESRD) in developed and developing countries. DKD in some parts of the world represents over 50% of patients requiring dialysis and/or transplantation. It is evident that hemodynamic and metabolic pathways interact to promote the development of DKD [3], as reflected by the clinical associations of systemic blood pressure and glycemic control with DKD. This review will highlight some of the molecular mechanisms and key pathways associated with susceptibility and progression of DKD. 

## 2. Vasoactive Pathways

### 2.1. RAAS Pathway

#### 2.1.1. Renin-Angiotensin-Aldosterone System (RAAS)

**Figure 1:**
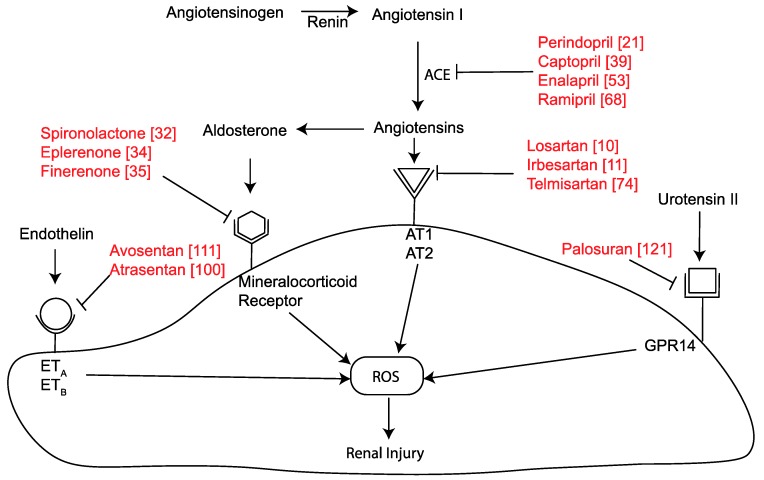
Role of vasoactive hormone pathways and relevant inhibitors in modulating renal injury via an effect on reactive oxygen species (ROS) [10,11,21,32,34,35,39,53,68,74,100,111,121].

The RAAS pathway is one of the best-studied vasoactive pathways (Figure 1). Historically, the RAAS was thought to be a major regulator of blood pressure, water, and electrolyte homeostasis [4,5]. However, over the last few decades, a role for the RAAS in regulating cell growth and differentiation, extracellular matrix (ECM) metabolism, and inflammation in chronic diseases has been reported [6,7,8,9]. Clinical trials of angiotensin-converting enzyme (ACE) inhibitors and angiotensin II (Ang II) receptor blockers (ARBs) supported the experimental findings and were shown to retard the progression of renal disease [10,11]. These studied established that the RAAS is a major player in the development and progression of DKD.

The canonical RAAS pathway begins with the production of angiotensinogen. First, renin converts angiotensinogen to angiotensin I (Ang I). Next, angiotensin-converting-enzyme ACE, also known as ACE-1, converts Ang I into angiotensin II (Ang II). The liver is a major source of circulating Ang I in mammals, but the expression of angiotensinogen has also been reported in other organs including the diabetic kidney [12,13] and the heart [14]. Ang II continues to be regarded as the primary effector molecule of the RAAS. Renin, aldosterone, and prorenin, the classical components of the RAAS, are now known to play major roles in DKD, making them attractive therapeutic targets (Figure 1). Important biological roles have also been discovered for other components of the RAAS, including metabolites of Angiotensin II, Ang (1-7) and Ang (1-9) and enzymes involved in Ang II synthesis and degradation, ACE2 and neprilysin [15]. These make up the alternative vasodilator RAAS pathway and are discussed later in further detail. 

Angiotensin II has several rapid effects, including constriction of the vascular tree, increased aldosterone secretion, the release of antidiuretic hormone, and increased myocardial contractility. In the glomerulus, Ang II increases intraglomerular pressure and induces alteration of glomerular basement permeability, promoting an increase in proteinuria [16]. Ang II also generates oxidative stress through NADPH (Nicotinamide Adenine Dinucleotide Phosphate) oxidase and acts as a promoter of inflammation and fibrosis, via activation of proinflammatory and prosclerotic cytokines [17]. Ang II primarily acts through two specific G-protein-coupled receptors, the angiotensin type 1 (AT1) and angiotensin type 2 (AT2) receptors. Both of these receptors are widely expressed and remain expressed at sites of diabetic complications [18]. The AT1, receptor subtype, is responsible for most of the physiological and pathological effects of Ang II. Interaction of Ang II and the AT1 receptor results in coupling of G proteins and generation of adenylyl cyclase, inducing many downstream pathways such as phospholipase C/inositol triphosphate/diacylglycerol/Ca2+ pathway, MAP kinases, tyrosine kinases and phosphatases, JAK signaling, NADPH oxidase, STAT pathways, and RhoA/Rho kinase[19]. In AT1 KO mice with induced diabetes, less renal injury develops. This is consistent with the pivotal role that the AT1 receptor has been observed to play in mediating diabetes-related renal injury [20]. AT1 receptor antagonists have demonstrated kidney protection in diabetic complications [17]. AT1 receptor inhibition reduces the production of fibrotic cytokines and ECM accumulation, improves renal albumin permeability via regulating cytokines such as vascular endothelial growth factor (VEGF), and alters podocyte structure and function via altered nephrin activity [21].

AT1 and AT2 receptors share only 34% sequence homology but both have a similar binding affinity for Ang II [22]. AT2 receptor expression is much lower than AT1 and may act as a functional antagonist to AT1 receptors by attenuating ROS -mediated damage generated by AT1 receptor action [23]. The role of the AT2 receptor in diabetic complications is not well defined, and contradictory results have been published. AT2 undergoes a complex tissue-specific regulation that may influence vascular development and repair [24]. A study using an AT2 antagonist as well as experiments in AT2 KO mice suggested that suppression of AT2 in diabetes leads to reduced macrovascular diseases [25] although renal effects remain controversial. Previous studies have also suggested that AT2 activation is linked to antiproliferative and anti-inflammatory effects, and apoptosis [26,27,28,29].

Aldosterone is released from the adrenal glands in response to various angiotensins including Ang II and III and in response to changes in serum potassium concentrations. Within the kidney, aldosterone acts as a hormone to stimulate reabsorption of sodium ions and water and release potassium ions into the urine for excretion. The downstream effects of aldosterone are propagated via activation of the mineralocorticoid receptor (MR). Aldosterone thus promotes water and salt retention to increase blood volume, which ultimately results in increased blood pressure [30]. In response to declining blood pressure, the adrenal gland stimulates aldosterone release and increases sodium reabsorption. An increase in sodium alters the extracellular osmolarity, which produces a complementary rise in systemic blood pressure. After long-term ACE inhibition or ARBs, many patients show an increase in aldosterone levels and this may be accompanied by ongoing renal damage [31,32]. In experimental models, a MR blocker reduces albuminuria, glomerulosclerosis, renal macrophage infiltration, renal monocyte chemoattractant protein 1 (MCP-1) synthesis, and expression of MCP-1′s upstream transcription factor NF-κB[33]. Although aldosterone blockade may be a potential therapeutic target in DKD, caution needs to be taken as MR blockers are associated with hyperkalemia [34]. Nevertheless, the combination therapy of aldosterone blockade with ACE inhibition or ARB may provide additional renoprotection in patients with DKD. This approach has been rekindled with the advent of new MR blockers with less hyperkalemia [35]. 

#### 2.1.2. Vasodilatory Arm of RAAS

ACE2, Ang-(1-7) and Ang-(1-9) are key components of the noncanonical RAAS pathway [15,36]. Like ACE, ACE2 belongs to the family of zinc metalloproteases. ACE2 is a membrane-bound enzyme that is also found in a soluble form in the plasma as well as in tissues such as the heart, liver, kidney, brain, and blood vessels [37]. ACE2 gene expression is high in the kidney and low in the heart, aorta, lung, and retina. ACE and ACE2 share 40% amino acid sequence homology but have different substrate specificities [38]. ACE2 is a mono-carboxypeptidase with a single active site and a greater (~400-fold) affinity for Ang II than Ang I [39,40]. Since ACE2 degrades Ang II to Ang (1-7), it has been postulated that ACE2 opposes ACE to regulate the balance between Ang II and Ang (1-7) [41]. Experimental studies report that the expression level of ACE2 in the diabetic kidney varies depending on the stage of the disease. In the streptozotocin-induced diabetic rat, ACE2 expression was shown to be decreased in the proximal tubules, while glomerular expression was increased [42]. In the db/db mouse model, ACE2 mRNA and protein expression were shown to be up-regulated in the renal cortex in early diabetes [43,44]. Based on these data it was hypothesized that in the early stages of DKD, ACE2 is up-regulated as a protective mechanism against the increase in ACE-dependent Ang II formation and subsequent development of DKD. As a result of prolonged hyperglycemia and consequent activation of proinflammatory and profibrotic pathways, ACE2 expression becomes down-regulated and this deficiency in the vasodilatory arm of the RAAS may contribute to disease progression. This hypothesis was supported by the study from Ye and colleagues, which showed that mice infused with an ACE2 antagonist developed albuminuria and glomerulosclerosis [44]. 

Ang-(1-7) can be produced from Ang I by different endopeptidases, by catabolism of Ang (1-9) by ACE, and from Ang II by ACE2 [45,46]. Ang (1-7), acts through the MasR receptor [47] and is a potent vasodilator shown to have antihypertensive, anti-inflammatory, and antiproliferative properties [46,48,49]. These effects suggest that Ang (1-7) acts as a counter regulator to AT1 receptor-mediated effects of Ang II. Ang-(1-7) can activate different effectors such as cyclooxygenase-2 or COX-2, forkhead box protein O1 (FOXO1), and vasodilator mediators such as prostanoids and NO (nitric oxide). An elegant study by Benter and colleagues [50] suggested that treatment with Ang-(1-7) and/or the Ang-(1–7) receptor MasR agonist AVE-0991 reduces albuminuria and abrogates the diabetes-induced abnormal vascular responsiveness to norepinephrine, endothelin-1, and Ang II. These observations suggest that Ang-(1-7) might be a renoprotective agent in diabetes. Ang-(1-7) also showed MasR-mediated cardioprotective effects via MAPK, phosphoinositide 3-kinase (PI3K)/Akt, and NADPH oxidase signaling pathways [51,52]. 

Ang-(1-9) was first detected in the early 1970s but was initially viewed to be biologically inactive and act indirectly by competing with Ang I for the active site of ACE [40,53,54]. The role of Ang (1-9) in DKD is not well understood, and the generation of Ang (1-9) from Ang II may only be relevant in the context of elevated Ang II [55]. However, there is increasing evidence to show that Ang-(1-9) has cardiovascular effects in vivo and in vitro via AT2 receptor activation [56,57,58]. It was recently shown that inhibition of the Rho-associated and coiled-coil-containing protein kinase signaling pathway increases ACE2 activity and plasma Ang-(1-9) levels [59]. Overexpression of various genes linked to vascular remodeling was normalized and mRNA eNOS (endothelial nitric oxide synthases) levels increased, revealing a novel role for Ang-(1-9) in vascular protection. Similarly, Ang-(1-9) infusion improved vasorelaxation and NO levels in spontaneously hypertensive stroke prone (SHRSP) rats. The same study also found that Ang-(1-9) increases NADPH oxidase 4 expression [60]. Nevertheless, further studies are needed to evaluate the significance of Ang (1-9) in the diabetic kidney.

#### 2.1.3. The RAAS Inhibition as a Therapeutic Target

The negative impact of AngII in DKD has been well documented and it is well accepted that ACE inhibitors (ACEi) and ARBs prevent or delay the progression of DKD in both type 1 and 2 diabetes [10,11,61,62]. The specific role of ACEi was first studied in type 1 diabetic subjects [62] and currently, it is a recommended therapy for the patient with type 1 diabetes, normal GFR but with an early stage of kidney damage and microalbuminuria [63]. 

The ALLHAT (Antihypertensive Lipid-Lowering Treatment to Prevent Heart Attack Trial) [64] and ADVANCE (Action in Diabetes and Vascular Disease: Preterax and Diamicron MR Controlled Evaluation) [65] trials have reported data on renal endpoints, including “hard” outcomes. In ALLHAT, an ACEi (lisinopril) was not better than a diuretic (chlorthalidone), or a calcium channel blocker (amlodipine) on the renal composite outcomes. The absence of a significant difference in ESRD between lisinopril and either chlorthalidone or amlodipine was confirmed after an extended follow-up. However, the data obtained in ALLHAT have limited significance due to the absence of any data on albuminuria and blood pressure control was different among treatment groups [64,66]. The composite renal outcome, in the ADVANCE trial, was significantly lowering the perindopril group than in the placebo group, with the results mainly explained by the positive effect on the new-onset of microalbuminuria, whereas the impact on doubling serum creatinine or ESRD was not significant [65,67]. In MICRO-HOPE (Microalbuminuria Cardiovascular and Renal Outcomes–Heart Outcomes Prevention Evaluation), DIABHYCAR, and the PERSUADE studies in T2D patients, no significant differences in the changes of serum creatinine or in renal outcomes were reported when comparing an ACEi with placebo [68,69,70]. However, in MICRO-HOPE [68] and DIABHYCAR [69], the rate of new-onset of persistent macroalbuminuria was significantly lower and the rate of regression from macro to micro or from micro to normal albuminuria was numerically higher in the ACEi group compared with the placebo group. Several meta-analyses (without differentiating between type 1 and type 2 diabetes) support the use of ACEi in diabetic patients with diabetic nephropathy, especially in the presence of significant albuminuria [71,72,73]. 

Evidence of the renoprotective role of ARB in T2D patients is more robust than for ACEi. The Irbesartan Diabetic Nephropathy Trial (IDNT) [11] and Reduction of Endpoints in NIDDM with the AII Antagonist Losartan (RENAAL) [10] trials included a majority of hypertensive patients with already advanced chronic kidney diseases (CKD) [10,11]. The IDNT compared irbesartan versus placebo versus amlodipine [11]. RENAAL compared losartan with placebo [10]. In both studies, the effect on lowering albuminuria was significantly better in the ARB groups and the risk of the renal composite outcome was significantly lower with the ARB compared with the placebo. 

The superiority of ARB compared to ACEis has been debated for a long time. In a direct comparison in randomized clinical trials in T2D patients with albuminuria, a similar effect on albuminuria and GFR decline was observed with telmisartan and enalapril [74]. The large ONTARGET trial, although not specifically dedicated to T2D patients, did not show any superiority in terms of renal endpoints or “hard” endpoints of telmisartan versus ramipril [75]. The pooled analysis also do not support any clear superiority of ARB over ACEis in T2D patients [71,72,76,77,78]. 

Because of two different modes of blocking the RAAS (inhibiting the conversion of Ang I to Ang II versus blocking the activation of the angiotensin type 1 (AT1) receptor subtype) and the residual risk of nephropathy progression on either treatment alone, combination treatments with ACEi and ARB were explored in order to achieve better RAAS blockade. This intervention showed promising results regarding blood pressure reduction and proteinuria [79,80], but one of the larger trials, Ongoing Telmisartan Alone and in Combination with Ramipril Global Endpoint Trial (ONTARGET), was terminated because of a lack of long-term benefits and a greater risk of acute renal failure and hyperkalemia, as compared to monotherapy with either agent [81]. Data have emerged from clinical trials demonstrating that the use of “supratherapeutic doses” (doses greater than those approved for lowering blood pressure), compared with standard doses, has favorable safety, tolerability, and efficacy in reducing proteinuria in both diabetic and nondiabetic patients with chronic kidney disease. Supratherapeutic dosing with one agent may be a valuable approach for optimizing RAAS blockade and providing renoprotection [82] rather than combination approaches using different RAAS blockers.

To achieve superior RAAS blockade, the use of a direct inhibitor of the upstream enzyme, renin was explored. The addition of the renin inhibitor aliskiren to the ARB losartan in type 2 diabetic subjects with nephropathy in the AVOID study led to an enhanced reduction in proteinuria despite minimal differences in blood pressure between groups [83]. However, the larger subsequent ALTITUDE (Aliskiren Trial in Type 2 Diabetes Using Cardiovascular and Renal Disease Endpoints) trial was terminated early because of a lack of benefit and potential harm with a signal toward an increase in a combined cardiovascular endpoint when compared to monotherapy with an ACEi or ARB [84] and a clear increase in hyperkalemia and hypotension. 

Another method of intensifying RAAS blockade involves the addition of a mineralocorticoid receptor antagonist (MRA) to ACEi or ARB, which has been shown to reduce albuminuria as well as inflammation and fibrosis in the kidney [85,86]. However, because of adverse side effects, this has limited its clinical utility especially in patients with impaired kidney function. The novel non-steroidal MRA finerenone appears to have a lower hyperkalemia risk despite having higher MR selectivity, potentially because of less renal accumulation than the older MRAs [87,88]. In patients with mild to moderate CKD and heart failure, finerenone reduced albuminuria and deterioration of renal function with a lower risk of hyperkalemia when compared to spironolactone [87]. In patients with type 2 diabetes, macroalbuminuria, and reduced kidney function addition of finerenone to ACEi or ARB treatment dose-dependently reduced albuminuria with only a 2–3% incidence of hyperkalemia [35]. Two-Phase 3 clinical trials (FIDELIO-DKD and FIGARO-DKD) are in progress to evaluate the effect of finerenone on nephropathy and cardiovascular disease in type 2 diabetic patients. 

### 2.2. Endothelins

The endothelin family consists of three endothelins: endothelin-1 (ET-1), endothelin-2 (ET-2), and endothelin-3 (ET-3). Endothelin-1, a 21 amino acid peptide, is considered the most potent, vasoconstrictor in the kidney with proinflammatory, mitogenic, and profibrotic properties [89,90,91,92]. ET-1 exerts its renal actions mainly through the two endothelin receptor subtypes, ETA and ETB [90,93,94]. In the kidney, ET-1 and its receptors are widely expressed in every cell type [94,95]. ET-1 appears to have a vital role in modulating renal function under normal physiological conditions and is an important mediator of pathophysiological conditions such as DKD. ETA and ETB receptor antagonists prevent the development of hypertension, renal vasoconstriction, inflammation, proteinuria, glomerulosclerosis, and tubulointerstitial fibrosis in experimental models of DKD [96,97,98,99,100]. The vasoconstrictive actions of endothelin-1 are primarily mediated via ETA receptors [93]. The ETB receptor is known to be involved in endothelin-1 clearance [101]. In various animal models, elevated expression of endothelin-1 has been observed in the diabetic kidney [98,101,102]. Experimental models of diabetic nephropathy have also shown increases in ETA receptor expression [102,103]. Under high glucose conditions in vitro, together with Ang II, increases in ET-1 mRNA and protein levels were observed [104,105]. Further, increased endothelin-1 increased ECM proteins via MAPK and PKC activation, and was associated with an increased concentration of ROS [104,106,107,108]. These findings support the view that renoprotection in the setting of diabetes may occur by antagonizing and/or its actions. Indeed, Heerspink et al. recently showed that the ET antagonist, Atrasentan reduced the risk of renal events in patients with diabetes and CKD. These findings from the SONAR trial suggest a role for selective endothelin receptor antagonists in protecting renal function in patients with T2Dat high risk of developing end-stage kidney disease [109]. These findings, build on a large number of studies with this [110] and other endothelin antagonists such as avosentan which have also demonstrated renoprotection [111]. 

### 2.3. Urotensin II

Urotensin II was first discovered nearly 40 years ago [112] and is considered the most potent vasoconstrictor identified to date [113]. However, its vasoconstrictive properties are restricted to certain regional vascular beds. Urotensin II is found primarily in the central nervous system but is also expressed at low levels in the kidney, spleen, prostate, thymus, adrenal glands, and small intestine and is also detected in blood and urine. It plays an important role in the pathogenesis of acute and chronic diseases, in stressful and adaptive reactions of the body, and in the development of pathological conditions such as renal failure and DKD [114].

Urotensin II has also been implicated in pancreatic beta-cell dysfunction and in diabetic retinopathy. Marked elevated levels of urotensin II and urotensin II receptor, also known as GPR14 expression have been observed in renal biopsies of patients with DKD [115,116,117]. This finding was further supported in animal experiments suggesting urotensin II/GPR14 as a mediator of renal fibrosis [118]. The molecular mechanism by which urotensin II promotes DKD is not well understood. Urotensin II causes ER stress and promotes the production of ECM in kidney tubular epithelial cells from diabetic mice, which leads to kidney fibrosis in diabetic nephropathy [116]. Increasing urotensin II expression in the kidney is associated with a significant increase in the synthesis of the profibrotic factors transforming growth factor-β1 (TGFβ1), fibronectin, and type IV collagen. Stimulation of the urotensin II receptor can also trigger vasodilation via an endothelium-dependent nitric oxide-mediated effect [119,120]. The role of urotensin II in kidney damage was confirmed in a clinical trial using the urotensin II receptor antagonist palosuran, which significantly reduced albumin excretion in diabetic patients with macroalbuminuria [121]. Totsune et al. [122] found that patients with type II diabetes and renal dysfunction demonstrated significant increases in urinary urotensin II, in proportion to their level of renal dysfunction. Elevated urinary and circulating urotensin II levels in diabetic patients with renal impairment have also suggested a possible role for urotensin II in the mediation of progressive renal disease [122]. A study by Tian et al. [118] demonstrated increased transcription of urotensin II and the urotensin II receptor in the kidneys of animals with experimental diabetes and further reported AngII and TGFβ1 as possible mediators of the increase in urotensin II gene transcription (Figure 1).

## 3. Metabolic Pathways

### 3.1. Mitochondria and Reactive Oxygen Species (ROS)

**Figure 2:**
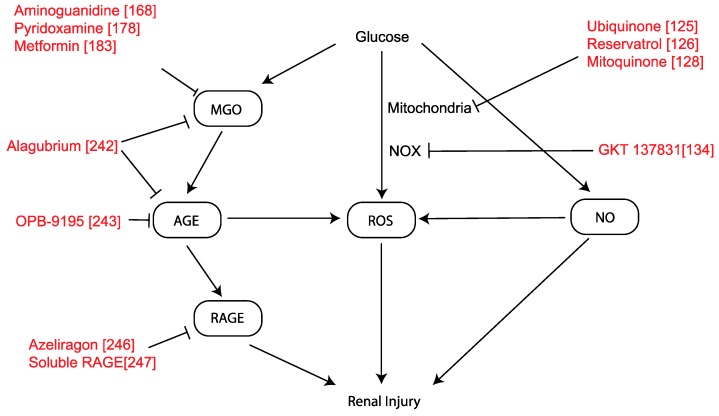
Role of glucose in promoting dicarbonyl and oxidative stress and reducing NO availability in order to promote renal injury [125,126,128,134,168,178,183,242,243,246,247].

The overproduction of ROS bridges the gap between altered metabolic pathways in the kidneys and disrupted renal hemodynamics known to be associated with DKD [123]. These pathways ultimately lead to inflammation, fibrosis, and endothelial dysfunction. Mitochondria produce energy in the form of adenosine triphosphate (ATP). This occurs via the electron transport chain, a series of proteins that work to create an electrochemical gradient using electrons sourced from fuels such as glucose, powered by hydrogen ion (H+) pumps, with energy harnessed to synthesize ATP and form water. When this process is dysregulated, such as in hyperglycemic states where glucose is in excess, it can result in excessive production of superoxide (O2−) and other ROS [2]. While ROS have a role in intracellular signaling, their accumulation can lead to oxidative stress, damage to critical cellular components (particularly protein and DNA), and cell death. Excessive ROS in the context of renal disease leads to renal fibrosis and a decline in renal function [124]. The damaging effect of ROS is thought to be mediated by activation of a number of pathways including PKC, NF-Kappa-B, hexosamine, and formation of advanced glycosylation end products (AGE). Physiological counter-regulatory mechanisms to protect against damage from ROS, in the form of antioxidants and repair enzymes have been explored. Antioxidants including coenzyme Q10 (ubiquinone), resveratrol, and ascorbic acid have been trialed in animal models of DKD with some evidence of therapeutic benefit [125,126,127] (Figure 2). Idebenone has preferential mitochondrial uptake by organs such as neurons, kidney, and cardiac tissues. Indeed, this compound is used in human respiratory chain diseases such as Friedreich ataxia where the mitochondrial generation of ATP appears to be preserved [128]. Similarly, mitoquinone has been trialed in mouse models of diabetes and showed improvement in glomerular function and albuminuria, potentially by promoting the destruction of defective mitochondria [128]. However, mitochondrial toxicity is a major concern in mitoquinone’s clinical utility [129]. Clinical trials using mitoquinone in CKD are underway and results are yet to be reported.

### 3.2. NADPH Oxidase (NOX)

NADPH oxidase (NOX) was originally discovered in neutrophils, where it produces vast quantities of O2− by electron transport to augment host-pathogen defenses [2]. In nonphagocytic cells, the ROS generated by NOX are postulated to act as second messengers. Activation of NOX can be triggered by several receptor-mediated pathways, such as receptors of AGEs, ANG II, and other cytokines and hormones [130,131]. Dysregulation of NOX activity is another source of oxidative stress contributing to the pathogenesis of DKD. NOX is also found in other cell types, where its physiological role is poorly understood but may involve signaling in the regulation of vascular tone and angiogenesis [124]. NOX-4 is a cytosolic and mitochondrial protein [132]. The potent NOX subunit NOX-4 is seen of increased levels in renal cells in animal models of DKD when compared to non-diabetic models [132]. Several studies suggest that upregulated NOX-4 is the primary source of ROS in the kidney, contributing to renal fibrosis and hence DKD [133].

In animal models, the deletion of NOX-4 and administration of a NOX-4/NOX-1 inhibitor GKT 137831 (Figure 2) have been shown to be renoprotective [134], but clinical trials in type 2 diabetic subjects did not show encouraging data albeit the trial duration was only for 3 months. Other NOX family members have also been explored. The NOX-1 isoform is considered to play a key role in atherosclerosis but a role in renal disease has not been confirmed [135]. Transgenic expression of NOX-5 in rodents, specifically in podocytes and mesangial cells, have demonstrated a likely pathogenic role for this isoform [136,137]. With NOX-2 playing a central role in immune defense it is critical that any NOX inhibitor is specific for the other isoforms without inhibiting NOX-2.

### 3.3. Nitric Oxide Synthase

Nitric oxide (NO) is a short-lived gas with biological and regulatory properties, produced via nitric oxide synthases (NOS). NO is a common free radical with a role in cellular signaling which is produced by numerous cell populations in mammals. Nitric oxide synthase (NOS) produces NO from NADPH, L-arginine, and oxygen. One of the major roles of NO is vascular dilatation following its release from endothelial cells. Indeed, NO is one of the most powerful vasodilators and is generally thought to be vasoprotective in the context of diabetes [138].

In diabetes, NOS is uncoupled following L-arginine depletion, where it produces superoxide instead of NO. This causes superoxide to accumulate at sites of diabetic complications [139]. Indeed, the administration of L-arginine to db/db mice prevents cardiac fibrosis [140]. There is, however, some controversy as to the contribution of NOS uncoupling to diabetic complications. Early in disease development, NO production within tissues is thought to increase [141] as a result of changes in NOS activity [139]. It has been postulated that the therapeutic blockade of this pathway could be beneficial at this stage of diabetes [142]. 

By contrast, most studies performed later in the progression of diabetes suggest that functional decline in complication-prone organs is seen in concert with a state of progressive NO deficiency [143]. These changes in NO production are attributed to multiple mechanisms such as glucose and AGE quenching, as well as inhibition and/or posttranslational modification of NOS. Indeed, several studies support this view, with chronic NO inhibition having been identified to have no effects [144] or detrimental outcomes for renal disease as a consequence of diabetes [145]. These complex temporal changes in NO production seen during the evolution of diabetic complications make it difficult to determine the clinical applicability of NOS activity inhibition, given that a deficiency in NO production seems to be an equally important pathological contributor to diabetic complications including DKD.

### 3.4. Dicarbonyl Synthesis

In diabetes, hyperglycemia leads to an increase in the accumulation of dicarbonyls in plasma and in cells because of increased formation or to decreased activity of the detoxifying system, or both. Increased dicarbonyl concentrations interfere with cellular homeostasis and this is referred to as dicarbonyl stress [146]. Dicarbonyl stress has been identified as a major contributing factor to the progression of diabetic complications. Methylglyoxal (MGO), glyoxal (GO), and 3-deoxyglucosone (3-DG) are the main dicarbonyls that are present in human plasma and cells [147]. Among these, MGO is the most reactive and abundant dicarbonyl present in the body and thus has gained the most attention. MGO is associated with hyperglycemia in diabetes, diabetic vascular complications, hypertension, dyslipidemia, and obesity [148]. 

MGO is mainly formed by the nonenzymatic degradation of the triose phosphates, glyceraldehyde-3-phosphate (G3P) and dihydroxyacetone-phosphate (DHAP), as a byproduct of glucose metabolism [149]. Under physiological conditions formation of MGO constitutes only 0.1% of the glucotriose flux [150]. MGO is highly reactive and only 1% of MGO exists in the free unhydrated, monohydrated or dehydrated form. MGO primarily reacts with the arginine residues of proteins [146]. Furthermore, MGO can react with genomic DNA which can lead to genomic instability [151]. 

Plasma levels of MGO are associated with the prevalence of CKD in diabetes [152,153,154]. Higher plasma MGO levels are associated with an increased risk for lower eGFR and increased albuminuria in both T1D and T2D [155,156,157]. In addition to impaired detoxification, low eGFR may contribute to higher plasma concentrations of MGO in patients with diabetic nephropathy because of metabolic stress in tissues which may enhance the formation of MGO and/or inhibit its metabolism [158]. 

Glyoxalase 1 (Glo1) plays an important role in maintaining MGO levels with impairment of the rate of MGO detoxification by Glo1 potentially determining susceptibility to diabetic nephropathy. The knockdown of Glo1 increases MG-H1 residues in proteins of renal glomeruli and tubules, accompanied by the development of albuminuria and mesangial expansion [159]. Overexpression of Glo1 in glomerular mesangial cells decreases glucose-induced expression of mitochondrial oxidative phosphorylation complexes I, II, and III, indicating that MGO plays a role in high glucose-induced oxidative stress in mesangial cells [160]. Glo1 overexpression in diabetic rats also decreases markers for nephropathy, such as urinary KIM-1. Diabetes-induced loss of podocytes in the glomerulus, one of the early hallmarks of diabetic nephropathy [161], is also attenuated by overexpression of Glo1 [162]. Therefore, MGO may contribute to albuminuria through the accelerated loss of podocytes. Similar results were obtained using kidneys from nondiabetic Glo1 knockdown mice, indicating that increased MGO alone is sufficient to cause kidney dysfunction [163]. 

A clinical study in type 2 diabetic patients reported a positive association of serum MGO levels with the urinary albumin/creatinine ratio (ACR) at baseline while changes in the estimated glomerular filtration rate were inversely associated with MGO during follow-up [157]. Similarly, levels of urinary and plasma MGO levels correlated with basement membrane thickness in two cohorts of patients while MGO levels in red blood cells were higher in progressors vs. non-progressors of diabetic nephropathy. This link between MGO and renal disease was confirmed in another study, which reported the correlation of plasma MGO levels with serum creatinine and ACR in type 2 diabetic patients [152]. 

Similar to MG-H1, the level of the 3-DG derived AGE, 3-DG-H1, are elevated in experimental diabetes in the renal glomeruli [164]. Plasma levels of 3-DG also correlate with glomerular basement membrane thickness [154]. Kusunoki et al. reported elevated serum levels of 3-DG in diabetic patients with normoalbuminuria and further elevations in those patients with microalbuminuria and overt proteinuria [165]. Compared to 3-DG and MGO-derived AGEs, the glyoxal-derived AGE G-H1 is present at rather low levels, except in plasma [164,166,167]. Finally, the glyoxal-derived DNA product, GdG was elevated in the plasma of diabetic patients which suggests that glyoxal in contrast to methylglyoxal is more relevant for the glycation of DNA rather than for protein glycation.

#### MGO as a Therapeutic Target

Reducing the accumulation of MGO can provide new therapeutic opportunities for minimizing the pathophysiological conditions associated with MGO stress. Approaches to limit MGO stress are based on (1) the direct quenching of MGO, (2) the prevention or reduction of the formation of MGO, and (3) compounds inducing the expression of the MGO degrading enzyme, Glo1. 

Aminoguanidine is effective in animal models of diabetes in lowering AGE formation possibly by quenching MGO and preventing diabetic complications including DKD [168,169] as well as retinopathy [170] and nephropathy [171]. Unfortunately, the use of aminoguanidine in two large clinical trials in individuals with T1D [172] and T2D [173] showed disappointing results and the trial was terminated. Interestingly, creatine shows structural analogy to aminoguanidine, and it has been shown that creatine is a scavenger for MGO under physiological conditions [174]. The dietary intake of creatine may thus provide a natural mechanism for the trapping of MGO. Experimental studies have shown that another drug that may quench MGO, alagebrium reduces large artery stiffness, left ventricular mass, diastolic stiffness of the heart, atherosclerosis, and diabetic nephropathy [175]. Clinical trials on alagebrium have shown mixed results. Two uncontrolled studies in hypertensive individuals have reported an improved aortic augmentation index and brachial artery flow-mediated dilation after alagebrium treatment [176,177]. Although the results from experimental studies were promising, several trials were prematurely discontinued because of financial reasons.

The natural vitamin B6 analog pyridoxamine has been described as an anti-glycating agent, which operates, at least partly, via the scavenging of MGO [178]. Clinical studies have shown variable data in phase II clinical trials. Pyridoxamine not only inhibited the formation of AGEs but also improved kidney function in T2D with overt nephropathy [179], whereas another trial found no beneficial effect of pyridoxamine on the reduction of serum creatinine levels [180]. 

Metformin is the most widely prescribed oral glucose-lowering agents for T2D, mainly due to the suppression of hepatic gluconeogenesis and increasing cellular uptake of glucose [181,182]. Metformin, similar in structure to the MGO scavenger aminoguanidine, is also able to trap MGO [183]. Indeed, it has been shown that metformin treatment reduces systemic plasma MGO levels [184,185], accompanied by increased levels of a metformin-MGO imidazolinone compound [186]. However, metformin has low MGO scavenging ability which suggests that scavenging of MGO might not be the primary effect by which metformin reduces systemic MGO concentrations [146] and this drug has not been shown to be renoprotective independent of its glucose lowering effect. However, one cannot exclude a potential renoprotective effect albeit only previously reported in an experimental study of a non-diabetic model of CKD [187].

Another treatment strategy for reducing MGO is the use of Glo1 inducers. Polyphenols are known to upregulate Glo1 expression [188,189,190]. Flavonoids are the largest group of polyphenols. It was reported, in a randomized, double-blind, placebo-controlled, crossover trial with pure flavonoids in healthy (pre)hypertensive men and women, that the polyphenol quercetin but not epicatechin decreased plasma MGO concentrations [191]. However, the effect could not be explained by an increase in the expression of Glo1, and therefore, the MGO-reducing capacity of quercetin is likely to be due to the scavenging activity of this drug [192].

Phenethyl isothiocyanate and sulforaphane [193] are compounds found in cruciferous vegetables that are known to activate nuclear factor erythroid 2-related factor 2 (Nrf2) [194]. Nrf2 plays an important role in protection against oxidative damage and the induction of antioxidant enzymes [195]. Activators of Nrf2 may be responsible for an increase in Glo1 mRNA, protein, and activity [193] levels, thus leading to a decrease in MGO levels [196]. Relatively low physiological concentrations of sulforaphane led to an increase in Glo1 expression [197] in lymphocytes. Studies found that trans-resveratrol (tRES) and hesperetin (HESP) coformulation is a potent inducer of Glo1 [198]. In a clinical trial, the tRES-HESP combination produced a 22% increase in the Glo1 activity of peripheral blood mononuclear cells together with a 37% decrease in plasma MGO [199]. Nevertheless, the renal effects of these various approaches to increase Glo1 have not been clearly described.

### 3.5. Advanced Glycation and RAGE

Advanced glycation of free amino groups on proteins and amino acids is a nonenzymatic post-translational modification, which begins with covalent attachment of heterogeneous sugar moieties. This reaction is influenced by many factors including intracellular glucose concentrations, pH, and time. There is a large body of evidence to show that advanced glycation may modulate insulin secretion [200] and signaling [201,202], stabilize ECM proteins via cross-linking and modify collagens including type IV collagen, a basement membrane glycoprotein [203,204,205]. 

Persistent hyperglycemia and oxidative stress accelerate the formation of AGEs [206]. In diabetes, not only do long-lived proteins become more heavily modified, but short-lived proteins are also altered by advanced glycation. In addition, glycolytic metabolites of glucose such as glyoxal and products of the Kreb’s citric acid cycle are much more efficient initiators of intracellular advanced glycation than glucose per se. AGE pathways are as heterogeneous as their products and occur as a result of complex biochemical reactions involving the formation of Amadori products, the pentose phosphate pathway glyceraldehyde-3-phosphate, and formation of the reactive carbonyl methylglyoxal, a dicarbonyl that was described earlier to play a role in diabetic complications [207].

The consequences of the modification of proteins by advanced glycation are numerous. Extracellular generation of AGEs has effects on matrix-matrix, cell-cell, or matrix-cell interactions. This has been shown under pathological conditions to excessively crosslink the matrix resulting in stiffening [208,209,210]. This may occur as a consequence of intracellular AGE modification of ECM proteins, altering their secretory properties and folding. 

AGEs can also interfere with cellular homeostasis via interaction with cellular receptors. There are many AGE receptors [211,212,213,214], but the role of the receptor for advanced glycation end products (RAGE) is the most widely studied in diabetic complications. RAGE is a pattern recognition receptor that binds to multiple ligands such as AGE modified proteins, HMGB1 [215], S100 calgranulins [216], and β-amyloid [216]. RAGE appears to have a major role in immune and inflammatory responses [217,218]. The ligation of AGEs to RAGE also results in NAD(P)H oxidase [219] and mitochondrial [220] dependent ROS generation. In diabetes, AGEs can induce the production of chemokines such as MCP-1 [221,222,223], profibrotic cytokines, and growth factors including TGFβ1 [224,225,226] and connective tissue growth factor (CTGF), and the angiogenic growth factor VEGF [205].

The *RAGE* gene can produce a number of protein splice variants [227,228,229] but membrane-bound and circulating RAGE are the most common RAGE products [229]. Circulating soluble RAGE can also be produced via cleavage of membrane-bound RAGE [230]. The capacity of soluble RAGE, a so-called decoy receptor, to compete for ligands appears to play an important role in the development and progression of diabetic complications. In diabetic individuals with complications, studies now conclusively show that increases in soluble RAGE are predictive of both cardiovascular events [231,232,233,234] and all-cause mortality [234,235]. 

Glycosylated hemoglobin level (HbA1c) is widely used as a marker of glycemic control and predictor of diabetic complications, albeit HbA1c is an earlier rather than an advanced glycation product. Moreover, studies in both T1D and T2Dconclusively show that elevation in HbA1c is one of the most useful prognostic indicators for risk in individuals with diabetes. Therefore, it is not totally surprising that elevations in circulating concentrations of RAGE ligands including AGEs [236] and HMGB1 [237] are predictive of macrovascular complications in diabetes. In addition, the urinary AGE concentration can act as a biomarker of DKD given that the ultimate fate of most AGE-modified proteins and peptides is getting excreted via kidney excretion [238,239,240].

In animal studies, B complex vitamins pyridoxamine (B6) and thiamine (B1) appeared to show evidence for reducing AGEs in preclinical studies, but have failed to show any major impact on DKD in clinical trials [241,242]. Alagebrium, which appears to have multiple actions including breaking crosslinks to dismantle AGEs as well as quenching MGO, has shown promising renal effects but when trialed in combination with an ACEi it did not confer additional renoprotection [243]. Further another AGE inhibitor, OPB-9195 [244] also can delay experimental diabetic nephropathy. 

Overexpression of glyoxalase-1 described earlier in this report is responsible for the removal of the AGE precursor MGO and this leads to a decrease in the tissue accumulation of AGEs [245,246]. A small-molecule RAGE inhibitor azeliragon has been trialed as a treatment in humans with Alzheimer’s disease as AGEs interact with beta-amyloid in the formation of plaques; however, it was unexpectedly found to accelerate cognitive decline [247]. This failure of a RAGE inhibitor resulted in the closure of that drug discovery program and ultimately was not tested in DKD. Administration of soluble RAGE or RAGE-neutralizing antibodies [248] in rodent models of diabetes have also shown protection against complications [219,220,249,250,251]. Although the reduction in AGEs or targeting RAGE remain promising approaches, because of the adverse effect in some cases possibly as a result of an intrinsic role for RAGE in innate and adaptive immunity [252,253,254] more careful pharmacological targeting of this pathway is required (Figure 2). 

## 4. Hemodynamic and Metabolic Pathway Interactions

As outlined previously, metabolic and hemodynamic pathways interact to promote DKD [3]. The underlying molecular mechanisms are not fully explained. There are often common mediators of injury as a result of the activation of either pathway. This includes ROS, signaling pathways such as protein kinase C (PKC) and activation of both profibrotic and proinflammatory pathways. Furthermore, there appears to be direct interactions between the two pathways. For example, Thomas et al. identified the effects of Ang II infusion in generating AGE whereas AGE infusion promoted expression of various components of the RAAS [255]. In another study, Fukami et al. demonstrated that AGEs could activate autocrine Ang II signaling in mesangial cells [256]. Recently a potential molecular mechanism linking the AT1 receptor to a key mediator of biological effects of AGEs, RAGE was identified. It was shown that RAGE transactivation mediates Ang II-induced inflammation. This occurs via the formation of a heteromeric complex of the AT1 receptor with RAGE [257]. The relevance of these receptor interactions remains to be fully elucidated within the kidney including in the setting of diabetes.

## 5. New Targets for Renoprotection

### 5.1. Sodium-Glucose Cotransporter 2 (SGLT2) Inhibitors

High-capacity, low-affinity SGLT2 transporters in the proximal tubules of the kidney are responsible for approximately 97% of the reabsorption of filtered glucose, thus minimizing glycosuria under normoglycemic conditions [258]. Sodium-glucose co-transporter-2 inhibitors are a unique class of anti-diabetic agents that have beneficial effects on blood pressure and body weight [259,260,261,262]. Under hyperglycemic conditions, the expression of SGLT2 in the proximal tubules is upregulated, thus increasing the threshold for glycosuria in diabetic subjects [263]. Pharmacological inhibition of SGLT2 (SGLT2i) reduces the capacity of the renal tubules to reabsorb glucose by at least 50%, thereby increasing glycosuria and lowering blood glucose levels [264]. The blood glucose-lowering efficacy of SGLT2i has been confirmed in both placebo-controlled and active comparator studies, and the additional benefits of weight loss, blood pressure reduction, and negligible risk of hypoglycemia have made SGLT2i popular as second-line therapy after metformin in T2D [258].

Experimental data with SGLT2i demonstrated reductions in intraglomerular pressure, proteinuria and histological manifestations of glomerular and tubular damage, even in the absence of blood pressure reduction [265,266]. In clinical trials, the initiation of SGLT2i treatment causes an acute, reversible decrease in eGFR [267,268]. Substantial reductions in cardiovascular morbidity and mortality, as well as hospitalizations for heart failure in cardiovascular outcome trials (CVOTs), are exciting additional benefits to SGLT2i therapy [269,270]. Subsequent long-term trials have reported sustained reductions in albuminuria and preservation of eGFR with these agents [267,271].

Remarkable benefits on the development and progression of nephropathy have not been entirely understood often in the context of marginal reductions in glycemia and blood pressure as achieved in some of the CVOTs [268,272]. It has been hypothesized that the upregulation and activation of SGLT2 in diabetes results in an increased proximal tubular reabsorption of sodium via the sodium-glucose cotransporter. The reduced sodium concentration at the macula densa level activates tubuloglomerular feedback (TGF) leading to increased intraglomerular pressure and hyperfiltration. Pharmacological SGLT2i reverses these pathophysiological changes, causing less sodium reabsorption in the proximal tubule, thus reducing sodium and fluid retention as well as systemic blood pressure with the increase in sodium and glucose concentration at the macula densa triggering adenosine release, which is a paracrine mediator of TGF downregulation [272,273]. Adenosine enhances arteriolar tone, resulting in reduced intraglomerular pressure, reduction in albuminuria, and amelioration of hyperfiltration. Increased hydrostatic pressure in Bowman’s capsule, because of the increased osmotic concentration of sodium and glucose, further enhances the effect of SGLT2i.

SGLT2i causes a diminished consumption of adenosine and oxygen in the proximal tubule, and thus reduces susceptibility to acute kidney injury (AKI) [268] as was seen in the EMPA-REG clinical trial [271]. Animal experiments have shown that SGLT2i downregulates inflammatory markers, oxidative stress and fibrosis [272]. A meta-analysis has suggested that SGLT2i have moderate benefits on atherosclerotic major adverse cardiovascular events but these benefits appear to be confined to patients with established atherosclerotic cardiovascular disease. However, SGLT2i have robust benefits in reducing hospitalization for heart failure and progression of renal disease regardless of existing atherosclerotic cardiovascular disease or a history of heart failure [274]. A primary renal trial, the Canagliflozin and Renal Endpoints in Diabetes with Established Nephropathy Clinical Evaluation (CREDENCE) study, was stopped prematurely because of positive renal outcomes [275]. The recently published results showed that in patients with T2Dand kidney disease, the risk of kidney failure and cardiovascular events was lower in the canagliflozin than in the placebo group.

### 5.2. Incretin-Related Therapies

Incretin-related therapies include dipeptidyl peptidase-4 inhibitors (DPP-4 inhibitors) and GLP-1 RAs (glucagon-like peptide type 1 receptor agonists). The GLP-1 peptide is a gastrointestinal hormone that acts as an incretin enhancing insulin secretion and has a pleiotropic effect on glucose metabolism. The incretin-based agents, GLP-1 RAs and DPP-4 inhibitors are novel antidiabetic drugs widely used as second-line therapy after metformin for the control of hyperglycemia in type 2 diabetic patients. GLP-1 RAs directly stimulate the GLP-1 receptor and DPP-4 inhibitors act by inhibiting the enzyme involved in the degradation of GLP-1, thereby increasing its serum concentration. Both drug classes exert their antihyperglycemic effect by stimulation of insulin secretion and suppression of glucagon secretion. In various rodent studies, incretin-based therapies decrease the activity of biomarkers of inflammation and fibrosis, urinary markers of oxidative stress, and glomerular leukocyte infiltration [276,277].

#### 5.2.1. GLP-1 Receptor Agonists

Currently, there are numerous GLP-1 analogues available. In the kidney, GLP-1 RA treatment induces a proximal tubular natriuresis through inhibition of sodium reabsorption by the sodium-hydrogen exchanger-3 [278]. Subsequently, GLP-1 RA treatment significantly increases the fractional excretion of sodium, to some extent resembling the effects seen with SGLT-2 inhibition [279,280]. Interestingly, GLP-1 RA agents do not seem to influence tubuloglomerular feedback and do not affect renal blood flow or GFR which was seen with SGLT-2i [281,282]. 

Without affecting renal hemodynamics, GLP-1 RA agents reduce albuminuria and renal morphological changes in animal models of diabetic nephropathy [283]. GLP-1RAs have also been shown to reduce inflammation, macrophage infiltration, oxidative stress, and the accumulation of type IV collagen in the kidney [278,284]. The SCALE diabetes trial, a randomized clinical trial designed to study the benefit of liraglutide on weight reduction, noted that the drug caused a dose-dependent reduction in albuminuria [285]. In the LEADER and SUSTAIN-6 trials, treatment with liraglutide or semaglutide was associated with a significant reduction in secondary renal endpoints, driven by a reduction in the progression to macroalbuminuria but no apparent effect on harder endpoints related to renal function such as preventing end-stage renal failure [286,287]. Similarly, the ELIXA (Evaluation of Lixisenatide in Acute Coronary Syndrome) trial showed a significant reduction in both new-onset macroalbuminuria and progression of existing macroalbuminuria, but no significant effect on eGFR or the risk of doubling of serum creatinine [288]. In another small randomized clinical trial, exenatide showed a statistically significant reduction in urine albumin, urinary TGFβ1 and type IV collagen when compared to the sulphonylurea glimepiride [289]. In the EXSCEL trial (Exenatide Study of Cardiovascular Event Lowering Trial), a reduction of new-onset macroalbuminuria was also reported in patients treated with once-weekly exenatide compared with placebo, without significant changes in microalbuminuria and ESRD [290].

Integrated data from nine phase 2 and 3 trials for the GLP-1 RA dulaglutide showed reductions in albuminuria with dulaglutide compared to placebo or insulin glargine, although eGFR did not differ between treatment groups in these studies [291]. The multi-center, AWARD-7 trial, comparing 1-year treatment with two doses of dulaglutide to insulin glargine in type 2 diabetic patients with moderate to severe renal impairment, has shown a reduced decline in eGFR over the study period in both dulaglutide groups compared to the glargine group [292]. Positive results with respect to hard renal endpoints were reported in the REWIND study, which compared treatment with dulaglutide 1.5 mg once weekly to placebo in type 2 diabetic patients with established cardiovascular disease or at high cardiovascular risk. 

In conclusion, most studies involving GLP-1 analogues show a favorable effect on albuminuria, although none of these trials studied renal endpoints as primary outcomes. Large randomized controlled trial targeting GLP-1 effects on pre-specified primary renal outcomes are required to enhance our understanding regarding the effects of this drug class on DKD as is now planned in the FLOW study (NCT03819153) with semaglutide.

#### 5.2.2. DPP-4 Inhibitors

In the kidney, DPP-4 inhibition causes a distal natriuresis but does not generally significantly influence renal hemodynamics [293,294]. However, there are some preclinical studies where effects on eGFR have been reported with DPP-4 inhibitors. It has been postulated that this to be related to effects on stromal cell-derived factor 1 (SDF-1) another molecule that is modulated by DPP-4 [295]. Experimental studies with different DPP-4 inhibitors have shown reduced albuminuria and improvement in renal morphological damage in various models of nephropathy [296,297]. Although not fully characterized reductions in oxidative stress and in the formation of AGEs are believed to be possible molecular mechanisms for DPP-4 inhibitors affording renoprotection [298]. Among the available DPP-4 inhibitors, (linagliptin, saxagliptin, alogliptin, and sitagliptin) linagliptin has been the most extensively analyzed with regards to DKD since this is the only DPP-4 inhibitor with minimal renal metabolism. 

In a pooled analysis of phase 3 trials in adults with T2Dand albuminuria, 6-months of treatment with the DPP-4 inhibitor linagliptin reduced albuminuria by 28% compared to placebo [299]. Moreover, in a pooled analysis of 13 trials, a 16% reduction in composite adverse renal events was observed [300]. However, in a randomized controlled trial, MARLINA-T2D, linagliptin treatment did not significantly reduce albuminuria compared to placebo [301]. Recently, the large CVOT known as CARMELINA reported neutral outcomes for the secondary renal endpoint (ESRD, renal death or 40% reduction in eGFR) but the reduction in albuminuria was confirmed [302]. In the other large (TECOS and SAVOR-TIMI 53) CVOTs with DPP-4 inhibitors, sitagliptin and saxagliptin reduced albuminuria significantly compared to placebo [303,304]. The EXAMINE trial did not find any difference in eGFR or incidence of initiation of dialysis with the use of alogliptin compared with placebo [305]. Harder renal endpoints did not show a significant difference between treatment groups in the SAVOR-TIMI 53 trial [303], whereas eGFR was significantly lower in participants assigned to sitagliptin in the TECOS trial [304]. Thus, in summary, DPP-4 inhibitors appear to have a beneficial effect on albuminuria but if this relates to primarily being glucose lowering agents remains controversial. 

Epidemiological data have indicated a link between anemia and progression of CKD in diabetes, but reduced hemoglobin is pathogenic or just reflects CKD is unknown [306]. Anemia is covariably associated with CKD and may be more severe for the same level of renal dysfunction in DKD [307]. Finally, the TREATS study used EPu to increase Hb in subjects with DKD but showed no benefit and indeed was associated with increased stroke [308].

## 6. Future Directions

Over the last two decades, there is an increasing body of data defining the molecular mechanisms responsible for the development and progression of DKD. With improved approaches to study the kidney such as single-cell sequencing, renal organoids, more sophisticated genomic and epigenomic approaches, and newer renal imaging techniques, more knowledge should become available on cell-specific changes in the diabetic kidney and molecular mechanisms of action by renoprotective drugs. With major progress over the last few years in reducing albuminuria and retarding a decline in GFR with some of the newer anti-diabetic agents, it is anticipated that the outlook for DKD will improve. With other new therapies mostly in the preclinical or early clinical phase, it is hoped that further breakthrough treatments will be identified either as a replacement or more likely as an adjunct treatment in diabetic subjects with or at risk of DKD.
